# Matrix Metalloproteinases Regulate the Formation of Dendritic Spine Head Protrusions during Chemically Induced Long-Term Potentiation

**DOI:** 10.1371/journal.pone.0063314

**Published:** 2013-05-16

**Authors:** Zsuzsanna Szepesi, Monika Bijata, Blazej Ruszczycki, Leszek Kaczmarek, Jakub Wlodarczyk

**Affiliations:** 1 Department of Molecular and Cellular Neurobiology, The Nencki Institute of Experimental Biology, Warsaw, Poland; 2 Neurobiology Center, The Nencki Institute of Experimental Biology, Warsaw, Poland; University of Iowa, United States of America

## Abstract

Dendritic spines are are small membranous protrusions that extend from neuronal dendrites and harbor the majority of excitatory synapses. Increasing evidence has shown that matrix metalloproteinases (MMPs), a family of extracellularly acting and Zn^2+^-dependent endopeptidases, are able to rapidly modulate dendritic spine morphology. Spine head protrusions (SHPs) are filopodia-like processes that extend from the dendritic spine head, representing a form of postsynaptic structural remodeling in response to altered neuronal activity. Herein, we show that chemically induced long-term potentiation (cLTP) in dissociated hippocampal cultures upregulates MMP-9 activity that controls the formation of SHPs. Blocking of MMPs activity or microtubule dynamics abolishes the emergence of SHPs. In addition, autoactive recombinant MMP-9, promotes the formation of SHPs in organotypic hippocampal slices. Furthermore, spines with SHPs gained postsynaptic α-amino-3-hydroxyl-5-methyl-4-isoxazole propionic acid (AMPA) receptors upon cLTP and the synaptic delivery of AMPA receptors was controlled by MMPs. The present results strongly imply that MMP-9 is functionally involved in the formation of SHPs and the control of postsynaptic receptor distribution upon cLTP.

## Introduction

Dendritic spines are small membranous protrusions that extend from neuronal dendrites. The majority of excitatory synapses in the mammalian brain are accommodated at the dendritic spines, representing the postsynaptic compartments of neuronal synapses. Dendritic spines usually consist of a spine head that is connected to the neuron by a thin spine neck. The spines exhibit considerable structural diversity and have been divided into distinct morphologic categories [1]. Their shapes include thin, filopodia-like protrusions (thin spines), short spines without a well-defined neck (stubby spines), and spines with a large bulbous head (mushroom spines). The morphology of dendritic spines is known to reflect their function. Dendritic spines are remarkably dynamic structures. Alterations in spine morphology and turnover are thought to play a major role in neuronal plasticity, including learning processes [2], [3].

Recently, spine head protrusions (SHPs) have attracted attention as a novel aspect of altered spine morphology that might contribute to functional neuronal network changes. Spine head protrusions have been shown to be filopodia-like processes from the spine head of mature pyramidal neurons [4]. The formation of SHPs was found to be triggered by altered neuronal activity and required α-amino-3-hydroxyl-5-methyl-4-isoxazole propionic acid (AMPA) receptor activation. Richards et al. (2005) demonstrated that SHPs were driven by iontophoretically applied glutamate, suggesting that SHP formation leads to activity-dependent changes in synaptic connectivity. Recently, Verbich et al. (2012) showed that, perisynaptic astrocyte remodeling and glutamate uptake are involved in SHP formation in hippocampal slices after incubation with tetrodotoxin (TTX) and glutamate iontophoresis. However, the molecular mechanisms that regulate the formation of SHPs remain elusive. The involvement of three components of tetrapartite synapses (i.e., pre- and postsynaptic neurons with astrocytic processes) may indicate that extracellular matrix (ECM) molecules are potential regulators of SHP development. Extracellular matrix molecules are regarded as the fourth component of the tetrapartite synapse as were shown to interact with synaptic components, including pre- and postsynaptic parts and glia, and they are able to modulate the activity of synaptic receptors and ion channels [5], [6].

Matrix metalloproteinases (MMPs) are a family of Zn^2+^-dependent endopeptidases that regulate ECM molecule turnover and the maintenance of tissue homeostasis in the developing and adult brain [7], [8]. Additionally, MMPs can liberate many peptides by the partial proteolysis of ECM macromolecules, thus regulating various cell activities [9], [10], [11]. Matrix metalloproteinases have attracted attention primarily in the context of central nervous system (CNS) disease and injury, but their importance in physiological processes in the CNS has also been appreciated [12], [13]. Several recent studies demonstrated the essential role of MMPs in learning and memory formation. MMP-9 (gelatinase B)-deficient mice displayed memory deficits [14]. Additionally, the use of an MMP-9 inhibitor prevented the maintenance of long-term potentiation (LTP), a cellular model of learning and memory [15], [16], whereas MMP-9 KO mice displayed late-LTP deficits and recombinant active MMP-9 restored LTP [14]. The function of MMP-9 in neuronal plasticity may be a consequence of the postsynaptic translation of MMP-9 mRNA and activity-dependent protein secretion [17], [18], [19]. However, the mechanisms by which MMPs contribute to changes in synaptic plasticity are not fully understood.

Recently, MMPs have emerged as novel regulators of dendritic spine morphology. Matrix metalloproteinases were found to be able to rapidly modulate synaptic structure and function through the shedding of synaptic adhesion molecules and cell surface receptors [20], [21], [22]. Although the effects of MMPs on spine morphology are influenced by the MMP concentration, treatment duration, and developmental stage of the neurons, these enzymes have the potential to alter spine structure [23], [24], [25], to potentiate glutamatergic transmission [14], [24], to increase *N*-methyl-D-aspartate (NMDA) receptor mobility at the synaptic surface [26], and to facilitate neuronal activity within in vitro networks of hippocampal neurons [27].

Herein, to investigate the impact of MMPs on the formation of SHPs, we employed a chemically induced long-term potentiation (cLTP) model according to a previously established protocol that is known to enhance neuronal network activity [27], [28]. We demonstrate that cLTP induced by forskolin, rolipram and picrotoxin increases endogenous MMP-9 activity at dendritic spines which regulate the formation of SHPs during cLTP in dissociated hippocampal cultures. Additionally, we show that spines that express SHPs gain AMPA receptors (AMPARs) in an MMP-dependent manner upon cLTP.

## Materials and Methods

### Ethics Statement

All experimental procedures were carried out in accordance with the Ethical Committee on Animal Research of the Nencki Institute, based on the Polish Act on Animal Welfare and other national laws that are in full agreement with EU directive on animal experimentation.

### Dissociated Hippocampal Cultures

Dissociated hippocampal cultures were prepared from postnatal day 0 (P0) Wistar rats as described below. Briefly, the brains were removed, and hippocampi were isolated on ice in dissociation medium (DM; 81.8 mM Na_2_SO_4_, 30 mM K_2_SO_4_, 5.8 mM MgCl_2_, 0.25 mM CaCl_2_, 1 mM HEPES [pH 7.4], 20 mM glucose, 1 mM kynureic acid, and 0.001% Phenol Red). Hippocampi were later incubated twice with papain solution (Worthington, NY, 32H13491, 100 U/10 ml) for 15 min at 37°C and subsequently rinsed three times in DM. Digestion was stopped by incubation with trypsin inhibitor solution (Sigma T9253, 100 mg/10 ml). Hippocampi were then rinsed in Modified Eagle Medium (MEM; 10% fetal bovine serum and 1% penicillin-streptomycin). Hippocampi were triturated in MEM until tissue clamps disappeared and the medium became cloudy. Triturated hippocampi were diluted 10 times in OptiMEM (Invitrogen) and centrifuged at 1,000×*g* for 10 min at room temperature. The resulting cell pellet was suspended in MEM. Cells were counted and plated at a density of 120,000 cells per 18 mm diameter coverslips (Assistant, Germany) coated with 1 mg/ml poly-D-lysine (Sigma) and 2.5 µg/ml laminin (Roche). Three hours later, MEM was replaced with 1 ml of prewarmed complete growth medium (Neurobasal-A without Phenol Red supplemented with 2% B-27, 1% penicillin/streptomycin, 0.5 mM glutamine, 12.5 µM glutamate, and 25 µM β-mercaptoethanol). The cells were kept at 37°C in 5% CO_2_ in a humidified incubator for 3 weeks. The cells were fed twice per week by replacing half of the culture medium. The cells were transfected with Effectine (Qiagen) or Lipofectamine 2000 Reagent (Invitrogen) according to the manufacturer’s protocol at 7–10 days in vitro (DIV) with a plasmid that carried RFP under a β-actin promoter. The experiments described below were performed on 18–23 DIV.

### Primary Cortical Cell Cultures

Cortical neurons were cultured from newborn (P0) Wistar rats. The rats were decapitated, and cortices were dissected on ice in DM (81.8 mM Na_2_SO_4_, 30 mM K_2_SO_4_, 5.8 mM MgCl_2_, 0.25 mM CaCl_2_, 1 HEPES [pH 7.4], 20 mM glucose, 1 mM kynureic acid, and 0.001% Phenol Red). Cortices were digested for 30 min in papain solution (Worthington, NY, 32H13491, 100 U/10 ml) at 37°C. The reaction was stopped by a triple wash in trypsin inhibitor solution (Sigma T9253, 100 mg/10 ml). The cells were seeded on 12-well plates covered with poly-D-lysine (50 µg/ml) at a concentration of 350,000 cells per well in Neurobasal-A medium (Invitrogen) supplemented with 2% B-27 (Invitrogen), 35 mM glucose (Sigma), 1 mM L-glutamine (Invitrogen), and 0.5% penicillin/streptomycin (Invitrogen). The cells were kept at 37°C in 5% CO_2_ in a humidified incubator for 14 days. The experiments were performed on 12–14 DIV.

### Cell Stimulation and Live Cell Imaging

A bath application of a mixture of forskolin, rolipram, and picrotoxin (all dissolved in DMSO) was applied to chemically induce LTP as described previously [28]. Cultured hippocampal and cortical neurons were incubated with 50 µM forskolin (Sigma), 0.1 µM rolipram (Sigma), and 50 µM picrotoxin (Sigma) in maintenance media for 40 min. To inhibit MMP activity, neuronal cultures were preincubated with 25 µM GM6001 (Millipore), a general MMP inhibitor, for 30 min at 37°C, and cLTP was then induced. As controls, cultures were treated with compound-free solvent (DMSO; Sigma). Live-cell imaging was performed with cultured hippocampal neurons on 18–21 DIV. To visualize neuronal dendrites and dendritic spines, neuronal cultures were transfected with a plasmid that carried RFP under a β-actin promoter on 7–10 DIV. Before imaging, the cells were mounted in an acquisition chamber in which the temperature and CO_2_ concentration were controlled. Living dendrites were imaged for 10–15 min before stimulation, and cLTP was then induced by bath application of forskolin, picrotoxin, and rolipram. Dendrite segments decorated with spines were imaged every 5 min for 40 min of cLTP. Special care was taken to check cell viability after the live-imaging sessions. Images were acquired using a Leica TCS SP 5 confocal microscope with a PL Apo 40×/1.25 NA oil immersion objective using 488 nm and 561 nm line of diode pumped solid state laser at 10% transmission (1024×1024 pixel resolution). A series of z-stacks were acquired for the cells at 0.4 µm step and an additional digital zoom that resulted in a sampling density of 0.07 µm per pixel. The sum of the z-stacks was analyzed using ImageJ software (National Institutes of Health, Bethesda, MD, USA), and the SHP density was calculated as the number of SHPs per µm. Three-dimensional reconstructions of confocal stacks were computed using Imaris software (Bitplane).

### Gelatinase Assay of Live Neuronal Cultures

To visualize gelatinase activity in live dissociated hippocampal cultures, cells were preincubated with fluorescein conjugate gelatin (40 µg/ml/well, DQ-gelatin, Molecular Probes) for 30 min at 37°C. This fluorescent substrate was quenched until digested by the gelatinases MMP-9 and MMP-2 in neuronal cultures. The increase in fluorescence was proportional to the proteolytic activity of gelatinases, which was visualized using a confocal microscope. Custom-written ColocalizationMagick software was used to quantitatively analyze the colocalization between gelatinase activity and neuronal dendrites. A quantitative colocalization map was generated according to the method introduced by [29]. The colocalization map contains the local information indicating the contributions of the regions of the image into overall colocalization measure.

### Live Staining of GluA1- and GluA2-containing AMPARs

To selectively label cell-surface GluA1- and GluA2-containing AMPARs, cells were incubated with antibodies against the extracellular domain of GluA1 (1∶100, rabbit, Enzo Life Sciences) and GluR2 (1∶200, mouse, Millipore) subunits for 15 min at 37°C in complete culture medium. The cells were later fixed with prewarmed 4% paraformaldehyde (PFA) for 15 min. After washing, the cells were blocked with 1% phosphate-buffered saline (PBS)-bovine serum albumin for 30 min. The samples were then incubated with secondary antibody conjugated to Alexa 488 (1∶500, anti-mouse) and Alexa-647 (1∶500, anti-rabbit) for 2 h at room temperature. After mounting, the samples were visualized under a confocal microscope (TCS, SP5, Leica) equipped with a 40×/1.25 NA oil immersion objective using a 488 nm Ar laser to excite Alexa 488 and 633 nm HeNe laser to excite Alexa 647 at a pixel resolution of 1024×1024 with a 5.4× optical zoom. The z-stacks of optical slices were acquired in 0.4 µm steps. The sum of z-stacks was analyzed using ImageJ software. The intensity of GluA1 and GluA2 immunostaining were determined using custom-written SpineMagick-Colocalization software. The main feature of the software is the detection of the contours of the dendritic spines and the separation of the spines from the dendrite. The software also allowed to calculate the compartmentalized colocalization, by choosing the user-defined approximate region of interest, and subsequently refining the region by segmenting the image inside the region of interest on the bases of fluorescence intensity (see also [30]).

### Blocking Dynamic Microtubules with Nocodazole

To inhibit dynamic MTs, cultured hippocampal neurons (21 DIV) were transfected with a plasmid that carried RFP under a β-actin promoter. The cultures were treated according to a protocol described previously [31]. Briefly, the cells were incubated with a low concentration of nocodazole (200 nM, Sigma) for 4 h at 37°C. At 3.5 hours of treatment, DQ-gelatin was added to the culture medium, and the cells were incubated for an additional 30 min. The cells were then mounted in an acquisition chamber in which the temperature (37°C) and CO_2_ concentration (5%) were controlled. cLTP was induced by injecting a mixture of forskolin (50 µM final concentration), rolipram (0.1 µM final concentration), and picrotoxin (50 µM final concentration) into the culture medium. Repetitive imaging of living cells (*n* = 7) was performed using a confocal microscope. Some hippocampal cultures were treated with 200 nM nocodazole for 4 h, and the medium was then discarded and exchanged to a nocodazole-free medium. The cultures were kept in nocodazole-free culture medium for 3 h at 37°C. cLTP was then induced, and the cells (*n* = 5) were visualized under a confocal microscope.

### Gel Zymography

After 10 and 40 min of cLTP, conditioned media were collected and mixed with 5×sodium dodecyl sulfate (SDS) sample buffer without β-mercaptoethanol. The samples were loaded on 8% SDS-polyacrylamide gels copolymerized with 2 mg/ml gelatin. After electrophoresis, the gels were washed twice in 2.5% Triton X-100 in PBS for 15 min and incubated in developing buffer (50 mM Tris-Cl [pH 7.5], 10 mM CaCl_2_, 1 µM ZnCl_2_, 1% Triton X-100 m, and 0.02% NaN_3_) on a shaking table for 48 h at 37°C. The gels were stained with Coomassie Brilliant Blue and briefly destained. The amount of active MMP-9 was quantified using the Syngene Ingenius gel documentation system (Syngene, Cambridge).

### Western Blot

After stimulation, the cells were lysed in lysis buffer that contained 20 mM Tris-Cl (pH 6.8) at 4°C, 137 mM NaCl, 25 mM β-glycerophosphate, 2 mM NaPPi, 2 mM ethylenediaminetetraacetic acid, 1 mM Na_3_VO_4_, 1% Triton X-100, 10% glycerol, 2 mM benzamidine, 1 mM phenylmethylsulfonyl fluoride, 0.5 mM dithiothreitol, and 10 µl/ml protease inhibitor mixture (Sigma). The protein concentration in each sample was measured using the BCA protein assay kit (Pierce). Lysates that contained an equal total amount of protein were mixed with 5×SDS sample buffer and denaturated by heating. A total of 20 µg of the protein samples was loaded on 12% SDS-polyacrylamide gels. After electrophoresis, the samples were electrotransferred onto polyvinylidene difluoride membranes (Immobilon-P, Millipore). The membranes were then blocked in 10% nonfat milk in Tris-buffered saline with 0.1% Tween 20. After blocking, the membranes were incubated at 4°C overnight with anti-β-dystroglycan (NCL-b-DG, 1∶500, Novocastra) and anti-glyceraldehyde-3-phosphate-dehydrogenase (GAPDH; MAB 374, 1∶2000, Chemicon) diluted in 10% nonfat milk in Tris-buffered saline with 0.1% Tween 20. The membranes were then incubated with peroxidase-labeled secondary antibody diluted 1∶10000 in 10% nonfat milk in Tris-buffered saline with 0.1% Tween 20 for 1 h at room temperature. After washing, ECL plus reagent (GE Healthcare) was used to detect horseradish peroxidase (HRP) on the immunoblots.

### Immunocytochemistry

Hippocampal neurons were fixed with prewarmed 4% PFA for 30 min at room temperature, washed with PBS, and blocked with 10% normal goat serum (NGS) in PBS with 0.1% Triton X-100 for 2 h. After washing, the cells were incubated with anti-Bassoon antibody (rabbit, 1∶1000, Synaptic System) and anti-Homer-1 antibody (guinea pig, 1∶500, Synaptic Systems). After washing, the samples were incubated with fluorescent (Alexa 488, Alexa 647) secondary anti-rabbit and anti-guinea pig antibodies (1∶500, Invitrogen) for 2 h at room temperature. After mounting, the samples were visualized under a confocal microscope (TCS, SP5, Leica) equipped with a 40×/1.25 NA oil immersion objective using a 488 nm Ar laser to excite Alexa 488 and 633 nm HeNe laser to excite Alexa 647 at a pixel resolution of 1024×1024 with a 5.4×optical zoom. The z-stacks of optical slices were acquired in 0.2 µm steps. The sum of z-stack was analyzed using ImageJ software.

### Statistical Analysis

The data are expressed as mean ± standard error of the mean (SEM). Datasets were tested using the two-tailed Student’s t-test and if the number of groups were larger than two one-way ANOVA was used. Statistical analyses were performed using Origin 8 software (Origin Lab Corporation), and values of *p*<0.05 (*), *p*<0.01 (**), and *p*<0.001 (***) were considered statistically significant.

## Results

### cLTP Elevates MMP-9 but not MMP-2 Activity

**Figure 1:**
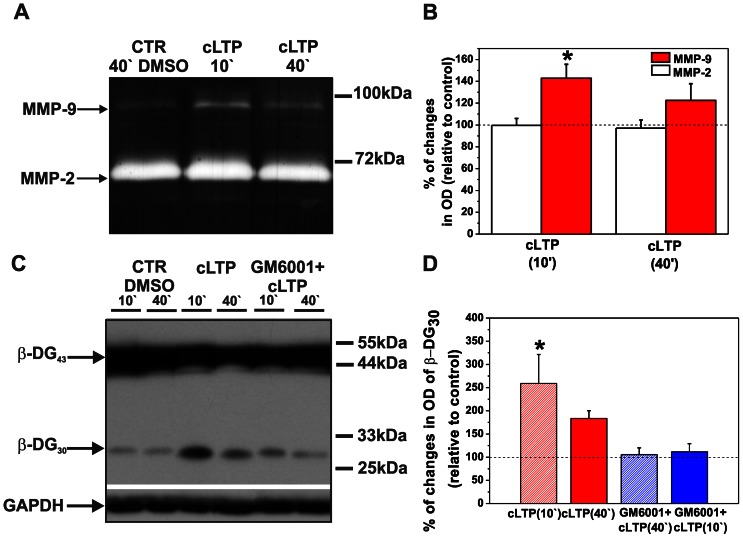
cLTP increases endogenous MMP-9 but not MMP-2 activity and increases β-DG cleavage by MMP-9. (**A**) Cortical neurons were exposed to the F/R/P mixture for 10 and 40 min, and gelatinase activity (MMP-9 and MMP-2) was then assayed. A representative gel zymogram shows enhanced MMP-9 activity compared with controls at 10 min, which was followed by a decrease at 40 min of cLTP stimulation. Notice that the enzymatic activity of MMP-2 did not change during cLTP. (**B**) Column bars present the quantification (mean ± SEM) of MMP-9 and MMP-2 activity 10 and 40 min after F/R/P treatment relative to control (DMSO-treated cells). Student’s t-test revealed a significant increase in the level of total amount of MMP-9 at 10 min. The level of MMP-9 did not significantly differ from controls after 40 min of cLTP. Student’s t-test did not reveal significant changes in the total amount of MMP-2 compared to control after 10 and 40 min of cLTP. (**C**) Cortical neurons were exposed to the F/R/P mixture, and β-DG cleavage was then tested by Western blot. A representative Western blot shows enhanced proteolysis of β-DG 10 min after stimulation compared with controls. Forty minutes of cLTP stimulation decreased the level of the cleaved form of β-DG. Blocking MMP activity with GM6001 led to a further decline of β-DG cleavage. Control cells were incubated with DMSO. Glyceraldehyde-3-phosphate dehydrogenase (GAPDH) served as the loading control. (D) Column bars indicate results of relative densitometric analysis of β-DG cleavage 10 and 40 min after F/R/P treatment relative to control (DMSO-treated cells). Students t-test showed statistically significant increase in cleaved form β-DG at 10 in of cLTP. Students t-test failed to reach significance for β-DG cleavage at 40 min of F/R/P exposure and for cLTP stimulation in the presence of MMP inhibitor, GM6001. Results are mean ± SEM, *n = *3, *p<0.05.

To examine the influence of cLTP induced by forskolin, rolipram, and picrotoxin (F/R/P mixture) on endogenous gelatinase (MMP-2 and -9) activity in cultured cortical neurons, gel zymography was performed. Cortical neurons were exposed to the F/R/P mixture, and then conditioned media were assayed at 10 and 40 min of cLTP. The time points were chosen to reveal the effect of endogenous MMP-9 activity on the spine morphology, according to the observations by Michaluk et al. (2011). Since the pro and active forms of MMP-9 were not clearly distinguishable on gel zymography and because the available antibodies are poorly specific towards rat MMP-9 and failed to differentiate pro- and active forms of the enzyme (that should be of 92 kDa and 88 kDa, respectively), we quantified total MMP-9 activity of 85–100 kDa wide band. As shown in Fig. 1A, F/R/P treatment led to a remarkable increase in the level of MMP-9 compared with control (i.e., Dimethyl sulfoxide (DMSO)-treated cells). This increase was found to be statistically significant at 10 min of treatment (relative optical density [OD_relative_] = 142.77±12.79, *p* = 0.016, *n* = 5, Fig.1B). Then, the level of MMP-9 activity decreased at 40 min of stimulation (OD_relative_ = 122.45±15.34, *p* = 0.281, *n* = 5). In contrast, the enzymatic activity of MMP-2 did not change upon cLTP stimulation (at 10 min: OD_relative_ = 99.56±23.91, *p* = 0.946; at 40 min: OD_relative_ = 97.160±24.595, *p* = 0.709). These results indicate that exposure to the F/R/P mixture transiently elevated MMP-9 activity in cultured cortical neurons, but with no effect on MMP-2 activity.

Michaluk et al. (2007) previously identified β-dystroglycan (β-DG) as an in vivo MMP-9 substrate in response to enhanced neuronal activity and showed that MMP-9 targeted 43-kDa β-DG to release a 30-kDa partial cleavage product. We tested whether the cLTP-induced upregulation of MMP-9 activity was associated with increased β-DG proteolysis. Cortical neurons were exposed to the F/R/P mixture for 10 and 40 min. Cell lysates were then collected, and Western blot analysis was performed. To verify whether ß-DG cleavage is MMP-dependent, cortical neurons were preincubated with the general MMP inhibitor GM6001 prior to cLTP stimulation. As shown in Fig. 1C, F/R/P treatment markedly increased the level of the cleaved form of ß-DG at 10 min, followed by a less evident increase at 40 min, as compared to controls treated with DMSO used as a solvent. Students t-test revealed a statistically significant increase in the amount of cleaved form of ß-DG at 10 min (OD_relative = _259.03±62.44, *p = *0.044, *n = *7, Fig. 1D) with no statistically significant difference at 40 min of cLTP stimulation (OD_relative = _156.78±28.27, *p = *0.575, *n = *3). Additionally, blocking the proteolytic activity of MMPs with GM6001 diminished β-DG cleavage in response to cLTP stimulation. Student’s t-test revealed no significant difference in β-DG cleavage when the cultures were preincubated with broad spectrum MMP inhibitor neither 10 nor 40 min after the cLTP stimulation (at 10 min: OD_relative = _105.53±14.45, *p* = 0.738, n = 3, at 40 min: OD_relative = _111.58±17.46, *p* = 0.575, n = 3, Fig. 1D). Thus, both gel zymography and increased β-DG cleavage provided evidence of the enhanced proteolytic activity of MMP-9 upon cLTP.

### cLTP Induces the Formation of Spine Head Protrusions

To investigate the dynamic mechanisms of the activity-dependent formation of SHPs upon cLTP, we used dissociated hippocampal cultures to facilitate the visualization and easier manipulation of individual dendritic spines. Dissociated hippocampal cultures were maintained for 21 days in vitro to allow the prominent accumulation of ECM molecules [32] and then were visualized under confocal microscopy.

**Figure 2:**
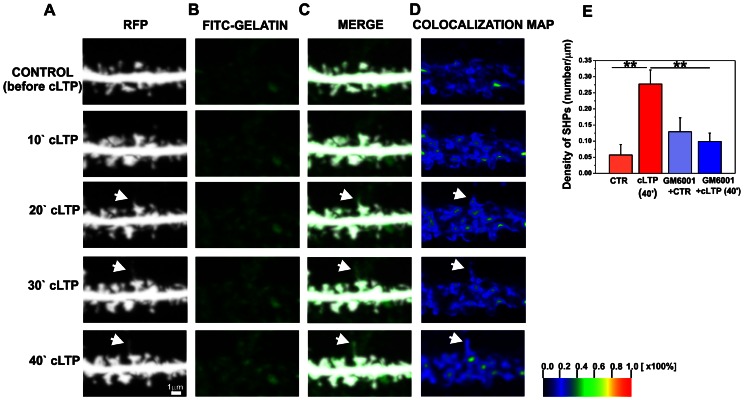
cLTP induces the formation of SHPs in an MMP-dependent manner. (**A**) Representative time-lapse imaging of secondary dendrite from pyramidal neuron expressing RFP is shown. (**B-C**) Cells were preincubated with FITC-gelatin to visualize gelatinase (MMP-9 and MMP-2) activity then were imaged until 40 min of cLTP stimulation. The time course of the appearance of a SHP (marked with arrow) indicates its formation 20 min after cLTP stimulation and it elongated till 40 min. (**D**) Colocalization maps show increased gelatinase activity at dendritic spine forming SHP (marked with arrow) at 20–40 minutes of cLTP. The colors on the colocalization maps correspond to the color-coded scale bar, indicating the percentage of colocalization and highlights pixels with high and similar intensities. (**E**) The bar plot shows the quantification (mean ± SEM) of the appearance of new SHPs (number/µm) on dendrites after 40 min of cLTP. Students t-test revealed statistically significant increase in newly formed SHPs after F/R/P stimulation compared to control. The inhibition of MMP activity by GM6001 blocked the development of SHPs in response to cLTP. Students t-test revealed significantly fewer newly formed SHPs in GM6001-pretreated cells than in the cLTP-stimulated group. CTR, before cLTP induction; cLTP, 40 min of cLTP; GM6001+ CTR, GM6001 pretreatment before cLTP induction; GM6001+ cLTP, GM6001 pretreatment and 40 min of cLTP. Results are mean ± SEM, n = 5, **p<0.01.

Live imaging of hippocampal neurons revealed that cLTP stimulation led to SHP formation (Fig. 2A). Spine head protrusions extended from the spine head and often exhibited terminal swelling. Spine head protrusions induced by cLTP were found to last for at least 40 min of cLTP stimulation, as followed throughout the live-cell-imaging session. The average length of SHPs observed was 1.30±0.079 µm (*n* = 31). Forty minutes of F/R/P treatment resulted in SHP formation in 10% of the spine profiles visualized. Importantly, SHPs at synaptic terminals were rarely observed in unstimulated dissociated hippocampal cultures.

Next, the contribution of MMPs to SHP development during cLTP was investigated. To visualize gelatinase activity in live hippocampal cultures, neurons were incubated with a fluorogenic substrate of gelatinases (fluorescein isothiocyanate [FITC]-labeled gelatin) during a live imaging session. Upon gelatinase activity induced by cLTP, the quenched fluorogenic substrates recovered, resulting in a fluorescent signal that was proportional to the amount of active enzymes. To localize gelatinase activity on neuronal dendrites, colocalization was quantitatively analyzed. Fig. 2 shows a short stretch of dendrites of hippocampal neurons (red fluorescent protein [RFP] channel, Fig. 2A) incubated with the fluorogenic substrate of gelatinases (FITC-gelatin, Fig. 2B) before and 10, 20, 30 and 40 min after cLTP stimulation. Time-lapse imaging of dendritic spines showed that SHP appeared as early as 20 min of cLTP with their length increasing till 40 min of stimulation. Additionally, Fig. 2D presents colocalization maps of imaged dendrites under control and cLTP-stimulated conditions. The detailed colocalization study confirmed that the appearance and elongation of SHP were associated with enhanced gelatinase activity at dendritic spines at 20–40 min of cLTP.

To investigate the contribution of MMPs to the formation of SHPs, live hippocampal neurons were incubated with GM6001 prior to cLTP stimulation. Images of neuronal dendrites were acquired before and 40 min after cLTP induction. The density of SHPs (number/µm) was quantified in cLTP-induced cells in absence or the presence of GM6001. One-way ANOVA revealed significant differences between groups of mean SHP density (p<0.001). Using Students t-test we found a statistically significant increase in SHP density (0.28±0.04 per µm, *p* = 0.004, *n* = 5 cells) at 40 min of cLTP compared with the unstimulated state (0.06±0.03 per µm; Fig. 2E). Blocking MMP activity with GM6001 significantly decreased SHP density (GM6001+ cLTP, 0.09876±0.02618, *p* = 0.010, *n* = 5 cells) as compared with cLTP-stimulated cells (0.28±0.04). Incubation with DMSO as an F/R/P solvent did not induce SHP formation (data not shown). Filopodia extensions that originated from dendrites were not included in our analyses. These observations demonstrate the MMP-dependent formation of SHPs and establish a role for MMPs in the remodeling of synaptic membranes during cLTP in cultured hippocampal neurons.

### Dynamic Microtubules are Involved in Spine Head Protrusion Formation during cLTP

**Figure 3:**
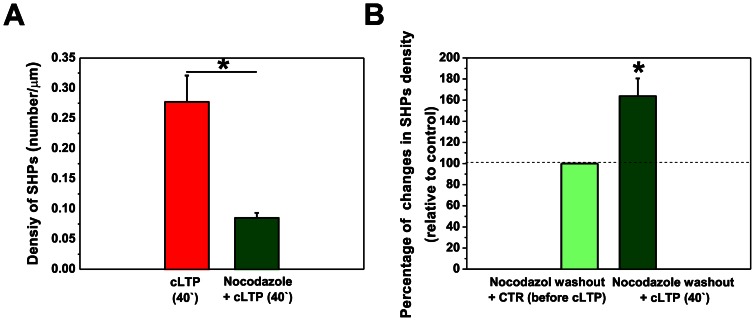
Dynamic microtubules are required for the development of SHPs upon cLTP. (**A**) Dissociated hippocampal cultures were preincubated with nocodazole (200 nM, for 4 h) to block neuronal microtubule dynamics and cLTP was then induced. Column bars show the quantification of SHPs (number/µm) under cLTP and nocodazole+cLTP conditions. Suppressing MT assembly/disassembly with nocodazole significantly reduced SHP density compared with the density measured after 40 min of cLTP. (**B**) Column bars show the quantification of SHPs (number/µm) in nocodazole washout experiments. The cultures were incubated with 200 nM nocodazole for 4 h, and nocodazole was then washed out. The cultures were kept in culture medium for 3 h, and cLTP was then induced. Blocking dynamic MTs with nocodazole followed by washout restored SHPs and significantly increased SHP density compared with unstimulated controls. The histograms show the mean ± SEM. *p<0.05.

Dynamic MTs have been found to transiently enter into dendritic spines, control spine morphology, and induce transient morphological changes by regulating actin dynamics [31], [33], [34], [35]. To determine whether neuronal MTs are required for the formation of SHPs during cLTP, MT dynamics were blocked by preincubation with nocodazole [31] prior to cLTP induction. Treatment with a low dose of nocodazole (200 nM) for 4 h prevented the formation of SHPs (0.09±0.009 per µm, Students t-test, *p* = 0.011, *n* = 7 cells) compared with the density of SHPs at 40 min of cLTP (0.28±0.043; Fig. 3A).

Hippocampal cultures were then incubated with 200 nM nocodazole for 4 h, and the medium was then discarded and changed to a nocodazole-free medium for 3 h and then cLTP was induced with the F/R/P mixture. Removing nocodazole from the medium restored SHPs after 40 min of cLTP (Fig. 3B). Student’s t-test revealed a statistically significant 163.75±16.81% increase in SHP density following cLTP compared with the unstimulated condition (*p* = 0.019, *n* = 5). Altogether, these observations demonstrated that dynamic MTs were necessary for SHP development and synaptic membrane remodeling during cLTP.

### Spine Head Protrusions can form Synapses

Time-lapse imaging of living dendrites revealed that cLTP stimulation induced SHP formation that could be identified as filopodia-like processes that protruded from the spine head. To determine whether spines that carry SHPs form synapses, hippocampal neurons were fixed 40 min after cLTP induction and immunocytochemistry was performed against postsynaptic Homer-1 and presynaptic Bassoon (i.e., markers of mature spines/synapses).

**Figure 4:**
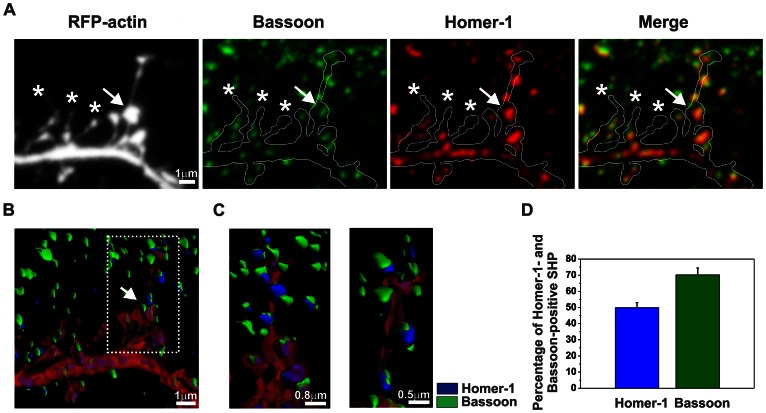
Spine head protrusions induced by cLTP form synapses. (**A**) A short segment of a neuronal dendrite labelled with anti-Bassoon and anti-Homer-1 antibodies is shown at 40 min of cLTP. Spine head protrusions with terminal swelling contained the postsynaptic marker Homer-1 and approached presynaptic Bassoon**.** The arrow indicates the location of a spine that turned into a SHP that exhibited terminal swelling. Short SHPs induced by cLTP did not exhibit terminal swelling and did not contain synaptic markers. Stars mark the location of spines that carried protrusions with a lack of synaptic markers. (**B-C**) Three-dimensional reconstruction of a short stretch of dendrite with spines and processes that emerged from the spine head after immunolabelling of presynaptic Bassoon (green) and postsynaptic Homer-1 (blue). (**B**) The arrow marks the formation of a SHP that exhibited terminal swelling. (**C**) High-magnification photographs depict SHP that contained postsynaptic Homer-1 and terminated at presynaptic Bassoon. (D) Column bars indicate mean (± SEM) percentages of Homer-1- or Bassoon-positive SHPs after 40 min cLTP.

Spines with SHPs expressed the postsynaptic marker Homer-1, which was aligned with presynaptic boutons (Bassoon-immunopositive). Long SHPs with terminal swelling were found to contain additional synaptic contacts at the end of the processes. These new synaptic contacts also possessed postsynaptic density (Homer-1-immunopositive, Fig. 4) and terminated at presynaptic boutons (Bassoon-immunopositive). We quantified the presence of synaptic markers in SHPs after 40 min of cLTP and measured as the percentage of Homer-1 or Bassoon-positive SHPs (Fig. 4D). We found that 49.81±3.25% of SHPs contain Homer-1 (*n = *16 cells) while 70.21±4.29% of SHPs were found to be apposed to Bassoon-positive puncta (*n = *16 cells). Additionally, we quantified the colocalization of Homer-1 and Bassoon at SHPs which revealed high correlation of the synaptic markers at SHPs (Pearson correlation: 0.533±0.020; *n = *16 cells). However, short SHPs (marked with stars in Fig. 4) lacked terminal swelling and did not form additional synaptic contacts at the tips of the processes.

These results demonstrated that SHPs induced by cLTP stimulation differed from filopodia or filopodia-like protrusions, which are usually characterized by a lack of postsynaptic density. These observations indicate the activity-dependent modification of synaptic connectivity during cLTP in dissociated hippocampal cultures. The proteolytic activity of MMPs is required for the formation of SHPs, providing further evidence of the involvement of MMPs in synaptic remodeling during plasticity.

### MMPs Control AMPAR Recruitment at Dendritic Spines that Express Spine Head Protrusions

**Figure 5:**
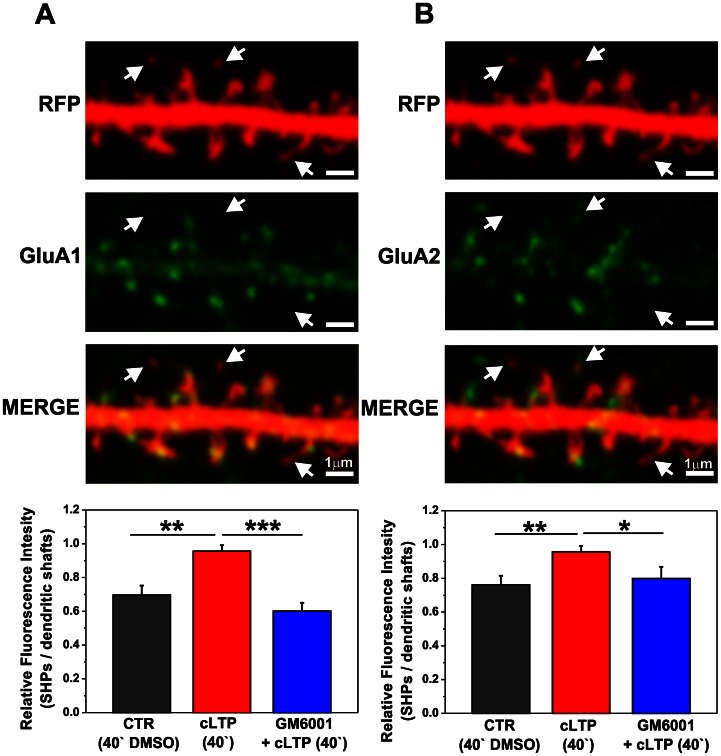
cLTP drives GluA1- and GluA2-containing AMPARs into spines that carry SHPs in an MMP-dependent manner. A short segment of a neuronal dendrite labeled with anti-GluA1 (**A**) and anti-GluA2 (**B**) antibodies is shown at 40 min of cLTP. Arrows indicate spines with SHPs. Bar plots show the quantification of fluorescence readouts from immunostaining against GluA1 (**A**) and GluA2 (**B**) subunits within spines that exhibited SHPs *vs*. dendritic shafts. (**A**). Students t-test revealed significant differences for GluA1-containing AMPAR content at spines with processes compared with control (DMSO-treated cells) after 40 min cLTP. Blocking MMP activity with GM6001 abolished GluA1-containing AMPARs surface delivery into spines with protrusions. (**B**) Forty minutes of cLTP induced the surface delivery of GluA2-containing AMPARs into spines with SHPs in an MMP-dependent manner. Students t-test showed significant differences for cLTP-stimulated cells compared with control and GM6001-pretreated groups. Results are mean ± SEM, n = 6, *p<0.05, **p<0.01, ***p<0.001.

Enhanced synaptic activity correlates with increased abundance of GluA1- and GluA2-containing AMPARs. Hence, we analyzed the abundance of those receptors at spines with SHPs. The extracellular domains of the GluA1 (Fig. 5A) and GluA2 (Fig. 5B) AMPAR subunits were immunolabeled in live hippocampal neurons expressing RFP. The relative distributions (i.e., fluorescent readouts that are proportional to the amount of GluA1 and GluA2 subunits) were calculated within dendritic spines with SHPs *vs*. dendritic shafts. One-way ANOVA of GluA1 immunostaining revealed differences between groups of control, cLTP-stimulated cells and cLTP-stimulated cells in the presence of GM6001 (p<0.001). We found that 40 min exposure of the cultures to the F/R/P mixture markedly increased the GluA1 immunostaining at spines with SHP *vs.* dendritic shaft (Fig. 5A). Student’s t-test revealed a statistically significant increase in the distribution of GluA1 subunits (0.92±0.137, *p* = 0.004, *n* = 6 cells) compared with controls (i.e., cell incubated with DMSO; 0.69±0.056, *n* = 6 cells). Blocking the proteolytic activity of MMPs with GM6001 significantly decreased the GluA1 enrichment of spines that carried SHPs (0.61±0.05, *p*<0.001, *n* = 6 cells) compared with GluA1 levels observed in cLTP-induced samples.

Furthermore, GluA2 immunostaining indicated a statistically significant increase in the ratio of spines with SHPs *vs*. dendritic shafts (Fig. 5B). A significant increase in spines with SHPs was found at 40 min of cLTP (0.96±0.04, *p* = 0.009) compared with controls that received DMSO treatment for 40 min (0.76±0.06). The relative amount of GluA2 AMPARs dropped to control levels after blocking MMP function with GM6001 (0.79±0.07, *p* = 0.050).

Altogether, these findings demonstrated that cLTP induced by the F/R/P mixture drove GluA1- and GluA2-containing AMPARs into spines with SHPs, and their accumulation in the synaptic membrane appeared to be MMP-dependent.

### Active MMP-9 Induces the Formation of Spine Head Protrusions in Organotypic Hippocampal Cultures

**Figure 6:**
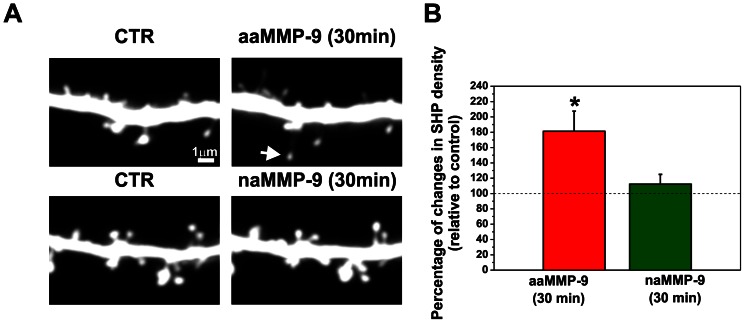
Enzymatic activity of MMP-9 is required for SHP development. (**A**) A segment of a secondary apical dendrite from a pyramidal neuron that expressed RFP was imaged with a confocal microscope (CTR) and then treated with autoactive MMP-9 (aaMMP-9) for 30 min. The white arrow shows a SHP formed after 30 min treatment with active MMP-9. Treatment with inactive MMP-9 did not lead to the formation of SHPs. (**B**) Bar plot shows the quantification of new SHPs per µm of dendrite. Students t-test revealed statistically significant increase in newly formed SHPs after treatment with active MMP-9. Incubation with inactive MMP-9 had no effect on the formation of SHPs Results are mean ± SEM, *p<0.05.

To determine the influence of MMP-9 on the formation of SHPs in a more complex model than dissociated hippocampal cultures, we performed live imaging of individual spines in organotypic hippocampal cultures. After transfection with an RFP plasmid using a biolistic method, CA1 area pyramidal neurons that expressed RFP were imaged by confocal microscopy. MMP-9 has been shown to exert some effects independently of its enzymatic activity [36], [37], therefore we also tested the effect of a non-enzymatically active form of MMP-9 (naMMP-9) on the dendrtitic spine morphology. Slices were treated with either recombinant aaMMP-9 or nonactive MMP-9 E402A [23], and images were acquired after 30 min of incubation (Fig. 6A). The density of SHP (number per µm) was calculated to determine the effect of active MMP-9 on SHP development. Student’s t-test revealed a statistically significant increase in the number of SHPs per µm after 30 min incubation with aaMMP-9 (181.296±26.254, *p* = 0.02121, *n* = 11) compared with controls (before treatment). No changes were observed in the density of SHPs in cultures treated with inactive MMP-9 for 30 min compared with controls (before treatment; Student’s t-test: 112.252±12.774, *p* = 0.4306, *n* = 8). These observations indicate that the proteolytic activity of MMP-9 and not simply protein-protein interactions is responsible for SHP development.

## Discussion

The present study demonstrated that cLTP induced by forskolin, rolipram, and picrotoxin increased endogenous MMP-9 activity and drove the formation of protrusions from dendritic spine heads in cultured neurons. The formation of SHPs was blocked by a broad-spectrum MMP inhibitor. Furthermore, we provided evidence that dynamic microtubules were required for the formation of SHPs during cLTP. Moreover, we showed that SHPs could form synapses, reflected by colocalization with pre- and postsynaptic markers. Additionally, we demonstrated that proteolytically active MMPs contributed to the synaptic recruitment of GluA1- and GluA2-containing AMPARs at spines with SHPs in response to cLTP.

Gel zymography and Western blot demonstrated that the cLTP stimulation of primary cortical cultures resulted in an elevated level of total MMP-9 compared with controls (i.e., DMSO-treated cultures). This effect was especially prominent 10 min after cLTP induction. Of note, since we could not separate clearly pro- and active forms of MMP-9 by the gel zymography method, we cannot exclude that also proMMP-9 displays enzymatic activity during cLTP due to conformational changes that led to remove the propeptide sterical hindrance at active site without direct proteolysis of propeptide [38]. Furthermore, we established a protocol that allows the detection of local net gelatinase (that may be produced by both MMP-9 and MMP-2) activity in live hippocampal cultures. Enhanced gelatinase activity was shown to localize on neuronal dendrites and dendritic spines. This experimental approach allowed us to demonstrate that SHP formation upon cLTP is associated with enhanced gelatinase activity. Additionally, we demonstrated that blocking MMP activity with GM6001, a broad spectrum MMP inhibitor, prevented SHP development.

The formation of SHPs represents a form of mature spine postsynaptic structural reorganization. Richards et al. (2005) previously demonstrated the occurrence of processes that “reach out” from spine heads to contact nearby presynaptic boutons and may result in multiple synapse boutons. Interestingly, SHPs were directly guided by iontophoretically applied glutamate. The authors hypothesized that SHP formation may represent changes in synaptic connectivity.

We demonstrated that SHPs with terminal swelling contained postsynaptic Homer-1, apposed to presynaptic Bassoon, indicating that SHPs induced by cLTP stimulation differed from filopodia or filopodia-like protrusions, which are usually characterized by a lack of postsynaptic density. In line with this finding, the presence of postsynaptic density at the tip of SHP induced by tetrodotoxin (TTX) was previously demonstrated by electron microscopy [4]. Of note, we also observed the formation of short SHPs lacking the postsynaptic marker. Similarly, dendritic spines that had nonsynaptic protrusions from the spine head were found in the CA1 area of hippocampus 30 min but not 2 h after LTP induction [39]. We hypothesize that short SHP without postsynaptic markers could represent a forerunner of SHP with postsynaptic density.

To further extend our observations, we also labeled cell-surface GluA1- and GluA2-containing AMPARs at dendritic spines that carried SHPs. The analyses of receptor abundance revealed that spines that expressed SHPs gained AMPARs after cLTP. Indeed, we showed that cLTP induced GluA1- and GluA2-containing AMPAR recruitment into spines with SHPs, and their synaptic incorporation appeared to be MMP-dependent. Blocking MMP activity with a broad-spectrum MMP inhibitor both abolished the formation of SHPs and eliminated GluA1 and GluA2-AMPAR delivery at the surface of spines with SHPs. It should be noted that GM6001 is a broad- spectrum MMP inhibitor which, besides MMP-9, is able to block other MMPs’ enzymatic activities that can be involved in changes of spine morphology. Our suggestion that MMP-9 might be the major enzyme involved in our experimental conditions comes from the findings that MMP-9 was the gelatinase upregulated during cLTP.

The precise mechanism by which MMPs regulate the cell-surface distribution of GluA1 and GluA1 AMPARs at dendritic spines is unclear. The Ephrin/Eph receptor complex has been identified as a substrate of MMPs, including MMP-9, at synapses during hippocampal LTP and shown to play a role in learning and memory [40], [41]. Interestingly, Ephs were shown to control glutamate receptor trafficking and clustering postsynaptically. Thus, one may speculate that MMP-9 contributes to the control of the synaptic incorporation of AMPARs through ephrin/Eph signaling during cLTP. However, further investigations are needed to clarify the role of MMPs in the synaptic recruitment of AMPARs during cLTP.

Our observations indicate that the formation of SHPs is related to changes in synaptic activity and the adaptation of neuronal connectivity during plasticity. Matrix metalloproteinases, predominantly MMP-9, contributed to the formation of SHPs and GluA1-and GluA2-AMPAR recruitment into spines with SHPs, providing evidence of the involvement of MMPs in synaptic connectivity alterations and the reorganization of neuronal networks during plasticity. SHPs are thought to be triggered by glutamate released from neighboring synapse and to form multiple synapse boutons by interacting with preexisting presynaptic contacts [4]. Multiple synapse boutons may contribute to spreading the information into the neuronal network and increase network activity by synchronizing the activity of the new synaptic contact with other neuron [42]. Interestingly, Niedringhaus et al. (2012) demonstrated that cLTP induced by forskolin and rolipram increased the network activity of cultured hippocampal neurons, an effect mediated by MMPs, predominantly MMP-9 and MMP-2, and β1-integrins.

The mechanisms by which MMPs induce postsynaptic structural reorganization and the formation of SHPs remain poorly understood and are likely to be numerous. Recent studies showed that MMPs can affect spine morphology through their ability to cleave cell adhesion molecules. Tian et al. (2007) demonstrated that activation of NMDA receptors induced cleavage of intercellular adhesion molecule-5 (ICAM-5), which was mediated by active MMP-9 and MMP-2. The shedding of ICAM-5 by MMP-9 and MMP-2 led to actin cytoskeleton reorganization and consequently the spine maturation of existing spines and elongation of filopodia. Furthermore, the ICAM-5 ectodomain was shown to interact with β1-integrins and stimulate the β1-integrin-dependent phosphorylation of cofilin, facilitating actin polymerization and spine maturation [21]. Additionally, the ephrin/Eph receptor complex is able to control the actin cytoskeleton [43], suggesting that MMP-9 mediates SHP formation via Eph signaling following its proteolytic cleavage.

Other MMPs may also contribute to neuronal activity dependent structural plasticity of dendritic spines. Indeed, recombinant MMP-7 alters the structure and function of presynaptic terminals [44], cleaves the NR1 subunit of NMDA receptors [45] and disrupts spine stability and induces spine morphological changes in vitro [25]. Additionally, MMP-3 was also demonstrated to cleave ICAM-5 and facilitate spine enlargement in association with LTP [21]. Furthermore, ADAM (a Disintegrin and Metalloproteinase) proteases, a family responsible for proteolytic degradation of the ECM proteins, were found to be involved in regulation of spine maturation and synaptic function, as well. Indeed, impairing ADAM10 localization and activity at synaptic sites enlarges spine head and increases the number and function of AMPA type glutamate receptors, both in vitro and in vivo [46], [47]. As GM6001 displays a broad spectrum activity against MMP as well as ADAM proteins, our observations do not exclude the contribution of other members of MMP and ADAM family in formation of SHP during cLTP. Future investigations are needed to further characterize the role of these proteases in remodeling of postsynaptic membrane and AMPAR recruitment during cLTP.

We also showed that neuronal MTs were required for SHP formation. Blocking dynamic pools of neuronal MTs abolished the development of SHPs induced by cLTP. Neuronal MTs have recently emerged as functional and structural regulators of dendritic spines by modulating the actin cytoskeleton [31], [33], [48]. Jaworski et al. (2009) demonstrated that growing MT plus-ends decorated by the microtubule tip-tracking protein EB3 could enter dendritic spines and were required to maintain their shape. Furthermore, these authors observed an interaction between MT-EB3 and p140Cap/SNIP, a regulator of Src tyrosine kinase that binds to cortactin (F-actin binding protein). Thus, the growth of EB3-labeled MT ends controls cortactin function and modulates actin dynamics in dendritic spines and subsequently spine morphology. Blocking dynamic MTs abolished the formation of SHPs, indicating that MTs are required for SHP formation, possibly because they can also control actin dynamics.

Moreover, the presence of MTs in spines may be associated with the formation of transient SHPs (tSHPs; [48]. However, tSHPS were found to be ephemeral structures, with an average lifetime of 40 s, suggesting that SHPs induced by cLTP, with a lifetime >20 min, are distinct structures. Because MTs are the main routes of transport of material from the cell body to dendritic spines, one may speculate that the postsynaptic protein redistribution required for SHP formation may at least partially depend on MT dynamics. Recently, Verbich et al. (2012) found that the formation of SHPs was accompanied by a reduction of overlapping astrocytic processes with dendritic spines, and blocking astrocytic glutamate transporters abolished SHP formation. Astrocyte remodeling and glutamate uptake are modulated by the Eph/ephrin interaction [49], [50], [51]. The authors suggested that Eph/ephrin communication is involved in SHP formation. These observations indicate that the formation of SHPs is regulated at multiple levels and involves glutamate receptor activation, perisynaptic astrocyte remodeling, the astrocytic control of glutamate spillover, and the contribution of dynamic MTs and proteolytic activity of MMPs.

Finally, we determined the influence of aaMMP-9 on SHP development in organotypic hippocampal cultures. We showed that aaMMP-9 triggered the formation of SHPs in organotypic hippocampal cultures. Furthermore, we provided evidence that the enzymatic activity of MMP-9 and not just protein-protein interactions is responsible for postsynaptic structural remodeling. Spine head protrusions appeared following 30 min treatment with aaMMP-9, and these kinetics are consistent with previous studies in which TTX incubation or iontophoretically applied glutamate triggered SHPs within 1 h [4], [52].

We propose that all of these observations may be interpreted in the following way. cLTP induced by forskolin, rolipram, and picrotoxin elevates the level of active MMP-9. Active MMP-9 cleaves some ECM components, adhesion molecules, cell surface receptors (e.g., ICAM-5 and β-dystroglycan) and triggers intracellular signals (e.g., through β1-integrins) that (*i*) induce the formation of SHPs that require actin reorganization controlled by dynamic microtubules and (*ii*) contribute to AMPAR recruitment into potentiated spines that carry SHPs.

In conclusion, the present data indicate the importance of MMPs in both the structural and functional aspects of synaptic plasticity. The data herein emphasize multiple roles of MMPs in synaptic plasticity, including postsynaptic structural remodeling and the control of postsynaptic receptor distribution.

## References

[1] Peters A, Kaiserman-Abramof IR (1970) The small pyramidal neuron of the rat cerebral cortex. The perikaryon, dendrites and spines. Am J Anat 127: 321–355. 4985058 10.1002/aja.1001270402

[2] Yuste R, Denk W (1995) Dendritic spines as basic functional units of neuronal integration. Nature 375: 682–684. 7791901 10.1038/375682a0

[3] Segal M (2002) Changing views of Cajal’s neuron: the case of the dendritic spine. Prog Brain Res 136: 101–107. 12143374 10.1016/s0079-6123(02)36011-4

[4] Richards DA, Mateos JM, Hugel S, de Paola V, Caroni P, et al (2005) Glutamate induces the rapid formation of spine head protrusions in hippocampal slice cultures. Proc Natl Acad Sci U S A 102: 6166–6171. 15831587 10.1073/pnas.0501881102 PMC556130

[5] Dityatev A, Rusakov DA (2011) Molecular signals of plasticity at the tetrapartite synapse. Curr Opin Neurobiol 21: 353–359. 21277196 10.1016/j.conb.2010.12.006 PMC3368316

[6] Dityatev A, Frischknecht R, Seidenbecher CI (2006) Extracellular matrix and synaptic functions. Results Probl Cell Differ 43: 69–97. 17068968 10.1007/400_025

[7] Mott JD, Werb Z (2004) Regulation of matrix biology by matrix metalloproteinases. Curr Opin Cell Biol 16: 558–564. 15363807 10.1016/j.ceb.2004.07.010 PMC2775446

[8] Sternlicht MD, Werb Z (2001) How matrix metalloproteinases regulate cell behavior. Annu Rev Cell Dev Biol 17: 463–516. 11687497 10.1146/annurev.cellbio.17.1.463 PMC2792593

[9] Nagase H, Woessner JF Jr (1999) Matrix metalloproteinases. J Biol Chem 274: 21491–21494. 10419448 10.1074/jbc.274.31.21491

[10] Ethell IM, Ethell DW (2007) Matrix metalloproteinases in brain development and remodeling: synaptic functions and targets. J Neurosci Res 85: 2813–2823. 17387691 10.1002/jnr.21273

[11] Michaluk P, Kaczmarek L (2007) Matrix metalloproteinase-9 in glutamate-dependent adult brain function and dysfunction. Cell Death Differ 14: 1255–1258. 17431423 10.1038/sj.cdd.4402141

[12] Rivera S, Khrestchatisky M, Kaczmarek L, Rosenberg GA, Jaworski DM (2010) Metzincin proteases and their inhibitors: foes or friends in nervous system physiology? J Neurosci 30: 15337–15357. 21084591 10.1523/JNEUROSCI.3467-10.2010 PMC3072038

[13] Huntley GW (2012) Synaptic circuit remodelling by matrix metalloproteinases in health and disease. Nat Rev Neurosci 13: 743–757. 23047773 10.1038/nrn3320 PMC4900464

[14] Nagy V, Bozdagi O, Matynia A, Balcerzyk M, Okulski P, et al (2006) Matrix metalloproteinase-9 is required for hippocampal late-phase long-term potentiation and memory. J Neurosci 26: 1923–1934. 16481424 10.1523/JNEUROSCI.4359-05.2006 PMC4428329

[15] Okulski P, Jay TM, Jaworski J, Duniec K, Dzwonek J, et al (2007) TIMP-1 abolishes MMP-9-dependent long-lasting long-term potentiation in the prefrontal cortex. Biol Psychiatry 62: 359–362. 17210139 10.1016/j.biopsych.2006.09.012

[16] Wojtowicz T, Mozrzymas JW (2010) Late phase of long-term potentiation in the mossy fiber-CA3 hippocampal pathway is critically dependent on metalloproteinases activity. Hippocampus 20: 917–921. 20572195 10.1002/hipo.20787

[17] Dziembowska M, Milek J, Janusz A, Rejmak E, Romanowska E, et al (2012) Activity-dependent local translation of matrix metalloproteinase-9. J Neurosci 32: 14538–14547. 23077039 10.1523/JNEUROSCI.6028-11.2012 PMC6621441

[18] Wilczynski GM, Konopacki FA, Wilczek E, Lasiecka Z, Gorlewicz A, et al (2008) Important role of matrix metalloproteinase 9 in epileptogenesis. J Cell Biol 180: 1021–1035. 18332222 10.1083/jcb.200708213 PMC2265409

[19] Konopacki FA, Rylski M, Wilczek E, Amborska R, Detka D, et al (2007) Synaptic localization of seizure-induced matrix metalloproteinase-9 mRNA. Neuroscience 150: 31–39. 17928157 10.1016/j.neuroscience.2007.08.026

[20] Michaluk P, Kolodziej L, Mioduszewska B, Wilczynski GM, Dzwonek J, et al (2007) Beta-dystroglycan as a target for MMP-9, in response to enhanced neuronal activity. J Biol Chem 282: 16036–16041. 17426029 10.1074/jbc.M700641200

[21] Conant K, Wang Y, Szklarczyk A, Dudak A, Mattson MP, et al (2010) Matrix metalloproteinase-dependent shedding of intercellular adhesion molecule-5 occurs with long-term potentiation. Neuroscience 166: 508–521. 20045450 10.1016/j.neuroscience.2009.12.061 PMC3535483

[22] Tian L, Stefanidakis M, Ning L, Van Lint P, Nyman-Huttunen H, et al (2007) Activation of NMDA receptors promotes dendritic spine development through MMP-mediated ICAM-5 cleavage. J Cell Biol 178: 687–700. 17682049 10.1083/jcb.200612097 PMC2064474

[23] Michaluk P, Wawrzyniak M, Alot P, Szczot M, Wyrembek P, et al (2011) Influence of matrix metalloproteinase MMP-9 on dendritic spine morphology. J Cell Sci 124: 3369–3380. 21896646 10.1242/jcs.090852

[24] Wang XB, Bozdagi O, Nikitczuk JS, Zhai ZW, Zhou Q, et al (2008) Extracellular proteolysis by matrix metalloproteinase-9 drives dendritic spine enlargement and long-term potentiation coordinately. Proc Natl Acad Sci U S A 105: 19520–19525. 19047646 10.1073/pnas.0807248105 PMC2614793

[25] Bilousova TV, Rusakov DA, Ethell DW, Ethell IM (2006) Matrix metalloproteinase-7 disrupts dendritic spines in hippocampal neurons through NMDA receptor activation. J Neurochem 97: 44–56. 10.1111/j.1471-4159.2006.03701.x PMC3369267 16515559

[26] Michaluk P, Mikasova L, Groc L, Frischknecht R, Choquet D, et al (2009) Matrix metalloproteinase-9 controls NMDA receptor surface diffusion through integrin beta1 signaling. J Neurosci 29: 6007–6012. 19420267 10.1523/JNEUROSCI.5346-08.2009 PMC6665240

[27] Niedringhaus M, Chen X, Dzakpasu R, Conant K (2012) MMPs and soluble ICAM-5 increase neuronal excitability within in vitro networks of hippocampal neurons. PLoS One 7: e42631. 22912716 10.1371/journal.pone.0042631 PMC3418258

[28] Otmakhov N, Khibnik L, Otmakhova N, Carpenter S, Riahi S, et al (2004) Forskolin-induced LTP in the CA1 hippocampal region is NMDA receptor dependent. J Neurophysiol 91: 1955–1962. 14702333 10.1152/jn.00941.2003

[29] Esposito A, Dohm CP, Kermer P, Bahr M, Wouters FS (2007) alpha-Synuclein and its disease-related mutants interact differentially with the microtubule protein tau and associate with the actin cytoskeleton. Neurobiol Dis 26: 521–531. 17408955 10.1016/j.nbd.2007.01.014

[30] Ruszczycki B, Szepesi Z, Wilczynski GM, Bijata M, Kalita K, et al (2012) Sampling issues in quantitative analysis of dendritic spines morphology. BMC Bioinformatics 13: 213. 22920322 10.1186/1471-2105-13-213 PMC3468369

[31] Jaworski J, Kapitein LC, Gouveia SM, Dortland BR, Wulf PS, et al (2009) Dynamic microtubules regulate dendritic spine morphology and synaptic plasticity. Neuron 61: 85–100. 19146815 10.1016/j.neuron.2008.11.013

[32] Karetko M, Skangiel-Kramska J (2009) Diverse functions of perineuronal nets. Acta Neurobiol Exp (Wars) 69: 564–577. 20048772 10.55782/ane-2009-1766

[33] Kapitein LC, Yau KW, Gouveia SM, van der Zwan WA, Wulf PS, et al (2011) NMDA receptor activation suppresses microtubule growth and spine entry. J Neurosci 31: 8194–8209. 21632941 10.1523/JNEUROSCI.6215-10.2011 PMC6622869

[34] Merriam EB, Lumbard DC, Viesselmann C, Ballweg J, Stevenson M, et al (2011) Dynamic microtubules promote synaptic NMDA receptor-dependent spine enlargement. PLoS One 6: e27688. 22096612 10.1371/journal.pone.0027688 PMC3214068

[35] Gu J, Firestein BL, Zheng JQ (2008) Microtubules in dendritic spine development. J Neurosci 28: 12120–12124. 19005076 10.1523/JNEUROSCI.2509-08.2008 PMC2605155

[36] Redondo-Munoz J, Ugarte-Berzal E, Terol MJ, Van den Steen PE, Hernandez del Cerro M, et al (2010) Matrix metalloproteinase-9 promotes chronic lymphocytic leukemia b cell survival through its hemopexin domain. Cancer Cell 17: 160–172. 20159608 10.1016/j.ccr.2009.12.044

[37] Ezhilarasan R, Jadhav U, Mohanam I, Rao JS, Gujrati M, et al (2009) The hemopexin domain of MMP-9 inhibits angiogenesis and retards the growth of intracranial glioblastoma xenograft in nude mice. Int J Cancer 124: 306–315. 18942717 10.1002/ijc.23951 PMC2814063

[38] Cauwe B, Opdenakker G (2010) Intracellular substrate cleavage: a novel dimension in the biochemistry, biology and pathology of matrix metalloproteinases. Crit Rev Biochem Mol Biol 45: 351–423. 20812779 10.3109/10409238.2010.501783

[39] Bourne JN, Harris KM (2011) Coordination of size and number of excitatory and inhibitory synapses results in a balanced structural plasticity along mature hippocampal CA1 dendrites during LTP. Hippocampus 21: 354–373. 20101601 10.1002/hipo.20768 PMC2891364

[40] Klein R (2004) Eph/ephrin signaling in morphogenesis, neural development and plasticity. Curr Opin Cell Biol 16: 580–589. 15363810 10.1016/j.ceb.2004.07.002

[41] Murai KK, Pasquale EB (2004) Eph receptors, ephrins, and synaptic function. Neuroscientist 10: 304–314. 15271258 10.1177/1073858403262221

[42] Harris KM (1995) How multiple-synapse boutons could preserve input specificity during an interneuronal spread of LTP. Trends Neurosci 18: 365–369. 7482800 10.1016/0166-2236(95)93930-v

[43] Ethell IM, Pasquale EB (2005) Molecular mechanisms of dendritic spine development and remodeling. Prog Neurobiol 75: 161–205. 15882774 10.1016/j.pneurobio.2005.02.003

[44] Szklarczyk A, Conant K, Owens DF, Ravin R, McKay RD, et al (2007) Matrix metalloproteinase-7 modulates synaptic vesicle recycling and induces atrophy of neuronal synapses. Neuroscience 149: 87–98. 17826919 10.1016/j.neuroscience.2007.07.032

[45] Szklarczyk A, Ewaleifoh O, Beique JC, Wang Y, Knorr D, et al (2008) MMP-7 cleaves the NR1 NMDA receptor subunit and modifies NMDA receptor function. Faseb J 22: 3757–3767. 18644839 10.1096/fj.07-101402 PMC2574025

[46] Malinverno M, Carta M, Epis R, Marcello E, Verpelli C, et al (2010) Synaptic localization and activity of ADAM10 regulate excitatory synapses through N-cadherin cleavage. J Neurosci 30: 16343–16355. 21123580 10.1523/JNEUROSCI.1984-10.2010 PMC6634827

[47] Gardoni F, Saraceno C, Malinverno M, Marcello E, Verpelli C, et al (2012) The neuropeptide PACAP38 induces dendritic spine remodeling through ADAM10-N-cadherin signaling pathway. J Cell Sci 125: 1401–1406. 22328515 10.1242/jcs.097576

[48] Hu X, Viesselmann C, Nam S, Merriam E, Dent EW (2008) Activity-dependent dynamic microtubule invasion of dendritic spines. J Neurosci 28: 13094–13105. 19052200 10.1523/JNEUROSCI.3074-08.2008 PMC6671621

[49] Murai KK, Nguyen LN, Irie F, Yamaguchi Y, Pasquale EB (2003) Control of hippocampal dendritic spine morphology through ephrin-A3/EphA4 signaling. Nat Neurosci 6: 153–160. 12496762 10.1038/nn994

[50] Nishida H, Okabe S (2007) Direct astrocytic contacts regulate local maturation of dendritic spines. J Neurosci 27: 331–340. 17215394 10.1523/JNEUROSCI.4466-06.2007 PMC6672072

[51] Nestor MW, Mok LP, Tulapurkar ME, Thompson SM (2007) Plasticity of neuron-glial interactions mediated by astrocytic EphARs. J Neurosci 27: 12817–12828. 18032653 10.1523/JNEUROSCI.2442-07.2007 PMC6673300

[52] Verbich D, Prenosil GA, Chang PK, Murai KK, McKinney RA (2012) Glial glutamate transport modulates dendritic spine head protrusions in the hippocampus. Glia 60: 1067–1077. 22488940 10.1002/glia.22335

